# MiR‐199a‐5p enhances neuronal differentiation of neural stem cells and promotes neurogenesis by targeting Cav‐1 after cerebral ischemia

**DOI:** 10.1111/cns.14323

**Published:** 2023-06-22

**Authors:** Hua‐Qian Jin, Wei‐Feng Jiang, Xin‐Tian Zheng, Lin Li, Yan Fang, Yan Yang, Xiao‐Wei Hu, Li‐Sheng Chu

**Affiliations:** ^1^ Department of Physiology Zhejiang Chinese Medical University Hangzhou China

**Keywords:** caveolin‐1 (Cav‐1), cerebral ischemia, miR‐199a‐5p, neural stem cells, neurogenesis

## Abstract

**Aims:**

MicroRNAs (miRs) are involved in endogenous neurogenesis, enhancing of which has been regarded as a potential therapeutic strategy for ischemic stroke treatment; however, whether miR‐199a‐5p mediates postischemic neurogenesis remains unclear. This study aims to investigate the proneurogenesis effects of miR‐199a‐5p and its possible mechanism after ischemic stroke.

**Methods:**

Neural stem cells (NSCs) were transfected using Lipofectamine 3000 reagent, and the differentiation of NSCs was evaluated by immunofluorescence and Western blotting. Dual‐luciferase reporter assay was performed to verify the target gene of miR‐199a‐5p. MiR‐199a‐5p agomir/antagomir were injected intracerebroventricularly. The sensorimotor functions were evaluated by neurobehavioral tests, infarct volume was measured by toluidine blue staining, neurogenesis was detected by immunofluorescence assay, and the protein levels of neuronal nuclei (NeuN), glial fibrillary acidic protein (GFAP), caveolin‐1 (Cav‐1), vascular endothelial growth factor (VEGF), and brain‐derived neurotrophic factor (BDNF) were measured by Western blotting.

**Results:**

MiR‐199a‐5p mimic enhanced neuronal differentiation and inhibited astrocyte differentiation of NSCs, while a miR‐199a‐5p inhibitor induced the opposite effects, which can be reversed by Cav‐1 siRNA. Cav‐1 was through the dual‐luciferase reporter assay confirmed as a target gene of miR‐199a‐5p. miR‐199a‐5p agomir in rat stroke models manifested multiple benefits, such as improving neurological deficits, reducing infarct volume, promoting neurogenesis, inhibiting Cav‐1, and increasing VEGF and BDNF, which was reversed by the miR‐199a‐5p antagomir.

**Conclusion:**

MiR‐199a‐5p may target and inhibit Cav‐1 to enhance neurogenesis and thus promote functional recovery after cerebral ischemia. These findings indicate that miR‐199a‐5p is a promising target for the treatment of ischemic stroke.

## Graphical abstract

Schematic diagram of the promotion of miR‐199a‐5p on endogenous neurogenesis via inhibiting of Cav‐1 signaling pathway during the recovery after ischemic stroke. Under physiological conditions, the miR‐199a‐5p/Cav‐1 signaling pathway promotes neuronal differentiation of NSCs. Low expression of Cav‐1 induced by miR‐199a‐5p contributes to the increased expression of BDNF and VEGF, eventually promoting neurogenesis.

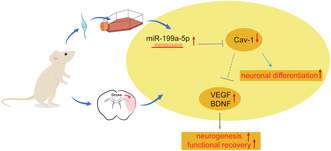

## 1 INTRODUCTION

Stroke as a common central nervous system (CNS) disease with high prevalence has been well established as a leading cause of disability and mortality.^1^ In particular, approximately 87% of cases can be further recognized as ischemic stroke.^2^ Recanalization, such as thrombolysis and endovascular treatment (EVT), are common treatment strategies for ischemic stroke,^3^ which, however, is limited by the narrow time range for therapeutic time window as well as by the risk of hemorrhagic transformation. Many patients thereby failed to receive such treatment in a timely manner.^4^, ^5^ It has been observed that the regenerative processes following a stroke, which may be inefficient though, persist actively in the CNS from days to weeks.^6^ An optimization of such regenerative process through promoting brain tissue repair therefore seems to be a promising approach to overcome the narrow therapeutic time window of stroke.^6^

Neural stem cells (NSCs) demonstrate such characteristic of self‐renewal and pluripotent differentiation that they can generate neurons, astrocytes, and oligodendrocytes in the nervous system.^7^, ^8^ In adult brains, NSCs are primarily located in two niches: the subventricular zone (SVZ) of the lateral ventricle and the subgranular zone (SGZ) of the dentate gyrus (DG) of the hippocampus.^9^, ^10^ Numerous preclinical and clinical studies have confirmed the ischemic insults activated endogenous neurogenesis, involving proliferation, migration, differentiation of NSCs, and integration into impaired neural networks.^11^ The stroke‐induced neurogenesis, however, is insufficient to restore full neurological function.^9^, ^12^ The majority of injury‐activated NSCs eventually differentiate into astrocytes. Transformation of the newly generated astrocytes into reactive ones in the injured brain contributes to the glial scar border and inhibits axon regeneration and neurogenesis.^13^ Exploring the molecular mechanisms of the stroke‐induced neurogenesis may thus facilitate the development of new treatment strategies for superior functional recovery.

MicroRNAs (miRNAs, miRs), which by increasing evidence have been closely related to the neuronal differentiation of NSCs and adult neurogenesis, are small noncoding RNAs that combine to the 3'untranslated region (3'UTR) of specific mRNAs and act as their post‐transcriptional suppressant.^14^ For one specific instance, miR‐199a‐5p has been detected in the CNS and participated in cerebral ischemic injury in rats.^15^, ^16^ It has been found in NSCs through high‐throughput sequencing approach a higher miR‐199a‐5p expression in ischemic SVZ than that in non‐ischemic SVZ, suggesting that miR‐199a‐5p may mediate neurogenesis after cerebral ischemia.^17^ Caveolin‐1 (Cav‐1),^18^ which hinders the differentiation of NSCs to neurons and oligodendrocytes and as well promotes the differentiation of NSCs to astrocytes,^19^, ^20^, ^21^ is one of the main target genes of miR‐199a‐5p, together with silent information regulator 1(SIRT1),^22^ hypoxia‐inducible factor 1α (HIF‐1α).^23^ However, whether miR‐199a‐5p promotes postischemic neurogenesis through targeted inhibition of Cav‐1 remains unclear.

In this study, it is aimed to identify whether miR‐199a‐5p/Cav‐1 pathway by enhancing endogenous neurogenesis promotes functional recovery after ischemic stroke.

## 2 MATERIALS AND METHODS

### 2.1 Isolation and culture of NSCs

Primary cultures of NSCs were separated from the cerebral cortex of rats at embryonic day (E) 14 as described previously^24^, ^25^ and then were cultured in DMEM/F12 (Gibco) supplemented with 2% B27 (Gibco), 20 ng/mL bFGF (PeproTech), 20 ng/mL EGF (Invitrogen), 1% penicillin–streptomycin. After 7 days of culture, NSCs were passaged by Accutase (Sigma‐Aldrich) with a ratio of 1:2.

### 2.2 Identification of NSCs

Firstly, the cells were immunostained with Nestin, a marker of NSCs, and then identified by proliferation and differentiation. For proliferation, single NSCs were cultured in poly‐d‐lysine‐coated plates. Twenty‐four hours later, the medium was changed with the 5‐bromo‐2′‐ deoxyuridine (BrdU) labeling solution (10 μM, Sigma‐Aldrich) for 48 h, and the cells were then immunolabeled for Nestin and BrdU. For differentiation, the cells were cultured in DMEM/F12 containing 1% fetal bovine serum (FBS, Gibco). After 7 days of differentiation, the cells were immunolabeled for neuronal, astrocytic, and oligodendrocytic markers (NeuN, GFAP, and Olig2, respectively).

### 2.3 Cell transfection

The miR‐199a‐5p mimic/inhibitor, Cav‐1 siRNA, and their corresponding negative controls (NC) were synthesized by RiboBio (Guangzhou Ribobio). Neurospheres were digested into single cells and seeded into 6‐well plates. Transfections of miR‐199a‐5p mimic (50 nM), inhibitor (100 nM), or Cav‐1 siRNA (100 nM) and relevant NC were conducted with Lipofectamine 3000 (Invitrogen) for 5 h, and then the medium was changed.

### 2.4 Dual‐luciferase reporter assay

The wild‐type (WT) or mutant (MUT) Cav‐1‐3′‐UTR vector and the pmirGLO luciferase reporter plasmid were synthesized by Promega (Promega). NSCs were transfected with pmirGLO‐WT or pmirGLO‐MUT and then cotransfected with miR‐199a‐5p mimic/NC using Lipofectamine 3000 for 5 h. The luciferase activity was measured 24 h after transfection with the dual‐luciferase reporter assay system (Promega). Results were normalized with Renilla luminescence.

### 2.5 Experimental animals

Male SD rats (280–300 g, SPF grade) were obtained from the Sino‐British SIPPR/BK Laboratory Animal (Shanghai, China). The rats were housed at 22 ± 2°C (a cycle of 12 h light/12 h dark). This study was authorized by the Experimental Animal Care and Ethics Committee of Zhejiang Chinese Medical University (Ethical approval No: ZSLL‐2015‐107).

### 2.6 Establishment of the middle cerebral artery occlusion (MCAO) model

The MCAO model was constructed as described previously.^26^ In brief, rats were anesthetized under isoflurane. After the right common (CCA) and external carotid artery (ECA) were isolated, the internal carotid artery (ICA) was exposed. A small opening was cut at the distal end of the ECA, and then a nylon monofilament suture with a tip diameter of 0.38 mm was advanced 18 ± 2 mm from the bifurcation of the carotid artery to block MCA origin. After 90 min of ischemia, the suture was withdrawn to restore the blood supply. Rats of the sham‐operation group underwent similar operations, but the nylon suture was inserted with a depth of 5 mm. The temperature of rats was controlled at around 37°C by a heating lamp during the whole surgical procedure.

### 2.7 Animal grouping and experimental treatment

Except for the sham group (*n* = 16), the rats with successful modeling were randomly divided into five groups: MCAO group (*n* = 26), MCAO+miR‐199a‐5p agomir (199a‐5p agomir) group (*n* = 26), MCAO+miR‐199a‐5p agomir NC (199a‐5p agomir NC) group (*n* = 26), MCAO+miR‐199a‐5p antagomir (199a‐5p antagomir) group (*n* = 26), and MCAO+miR‐199a‐5p antagomir NC (199a‐5p antagomir NC) group (*n* = 26). All rats were intraperitoneally administered with BrdU (50 mg kg^−1^ d^−1^, Sigma‐Aldrich) 24 h after MCAO for 14 days to label the proliferating cells. On day 5 after MCAO, miR‐199a‐5p antagomir/NC or miR‐199a‐5p agomir/NC were delivered into the lateral ventricle. Rats in the sham group and MCAO group were delivered with a corresponding volume of artificial cerebrospinal fluid (aCSF).

### 2.8 Intracerebroventricular injection

The intracerebroventricular injection was conducted following a previous report.^27^ In brief, the rats were anesthetized and set in a stereotaxic apparatus. The coordinates of intracerebroventricular injections were as follows: AP: −0.8 mm, L: −1.4 mm, V: −4.8 mm, approved by the stereotactic atlas of the rats. MiR‐199a‐5p agomir/NC (RiboBio, 5 μL/2.5 nmoL, dissolved in aCSF) and miR‐199a‐5p antagomir/NC (Ribobio, 5 μL/10 nmoL, dissolved in aCSF) or sterile aCSF (5 μL) were then slowly injected into the lateral ventricle at a speed of 0.25 μL/min at day 5 following MCAO. Then, the needle was maintained for an additional 10 min, followed by slow retraction.

### 2.9 Neurological function evaluation

Neurological deficit assessments were detected by researchers who were blinded to the design of experiments. All rats were tested for the modified neurological severity score (mNSS) and the corner test to evaluate neurological disorders on days 1, 3, 7, and 14 following MCAO. The mNSS is an 18‐point scoring system (0, normal; 18, maximal deficit).^28^ The corner test was applied to determine the sensorimotor function by counting the number of turns to the right of 10 trials.^29^

### 2.10 Detection of the volume of cerebral infarction

Rats were sacrificed and were perfused with saline followed by 4% paraformaldehyde (PFA) on day 14 after ischemia. The brains were fixed and dehydrated for frozen sections slicing. A 20 μm coronal section of brain tissue was immersed in 1% toluidine blue (Sigma‐Aldrich), followed to be dehydrated by gradient alcohol. The following equation measured the infarct volume percentage: volume of cerebral infarction (%) = (contralateral hemisphere volume − non‐infarct ipsilateral hemisphere volume)/contralateral hemisphere volume × 100%.

### 2.11 Immunofluorescence staining

For immunofluorescence staining for cells, the cells were fixed with 4% paraformaldehyde (PFA) for 15 min. Cells were then blocked for 1 h (10% goat serum) after permeabilizing with 0.1% Triton X‐100. For BrdU immunofluorescence, the cells were placed in HCl (2 moL/L) for 30 min, then neutralization with boric acids (0.1 moL/L) for 10 min, and then 3% H_2_O_2_. After that, cells were placed into primary antibodies of Nestin (1:200, Sigma‐Aldrich), BrdU (1:200, Sigma‐Aldrich), NeuN (1:200, Millipore), GFAP (1:200, Santa Cruz Biotechnology Inc.), and Olig2 (1:50, R&D Systems) overnight. After washes (3 × 5 min), cells were placed into the corresponding secondary antibody at 37°C for 1 h.

For immunofluorescence staining for brain slices, sections were post‐fixed with 4% PFA for 0.5 h and incubated with 2 × saline‐sodium citrate buffer supplemented with 50% formamide for 2 h at 65°C and placed in HCl (2 moL/L) for 0.5 h at 37°C. After being washed 3 times, the sections were neutralized with boric acids (0.1 moL/L) and then permeabilized, followed by 3% H_2_O_2_ at 37°C for 20 min. The tissue sections were then blocked for 1 h (5% goat serum), sequentially incubated with primary antibodies of BrdU (1:100), doublecortin (DCX, 1:100, Cell Signaling Technology), NeuN (1:500), and GFAP (1:100, Cell Signaling Technology) and then the corresponding secondary antibodies. The number of positive cells was counted from three sections per rat. Three fields were randomly selected from each section, and the average number of positive cells was calculated. All analysis was conducted with the examiner blinded to the identity of the samples being studied.

### 2.12 Quantitative reverse transcription‐polymerase chain reaction (RT‐qPCR)

The total RNA was isolated with Trizol reagent (Invitrogen), and the reverse transcription reaction was conducted with Mir‐XTM miRNA First‐strand Synthesis Kit (TaKaRa) for miRNA and PrimeScript RT Master Mix (TaKaRa) for mRNA. The real‐time quantitative PCR (qPCR) was performed with a TB Green Premix Ex Taq Kit II (TaKaRa) or TB Green Premix Ex Taq Kit (TaKaRa) on the Real‐Time PCR instrument (Applied Biosystems® 7500, Thermo Fisher Scientific). The results were calculated using the 2^−ΔΔCT^ method. The primers for RT‐qPCR are listed in Table 1.

**TABLE 1:** Primers used for the RT‐qPCR.

Target gene	Primer sequence (5′‐3′)
rno‐miR‐199a‐5p	GCCCAGTGTTCAGACTACCTGTTC
U6	
Forward	AGAGAAGATTAGCATGGCCC
Reverse	ATCCAGTGCAGGGTCCGAGG
Cav‐1	
Forward	CTACAAGCCCAACAACAAGGC
Reverse	AGGAAGCTCTTGATGCACGGT
GAPDH	
Forward	GCCAAGGCTGTGGGCAAGGT
Reverse	TCTCCAGGCGGCACGTCAGA

### 2.13 Western blotting

The total proteins were extracted using RIPA lysis buffer and measured with a BCA assay kit. Proteins were resolved by SDS‐PAGE. After blocking, the blots were incubated with the primary antibodies: anti‐Cav‐1 (1:1000, Cell Signaling Technology), anti‐NeuN (1:1000), anti‐GFAP (1:1000), anti‐GAPDH (1:1000, Huaan Biotechnology Co.), anti‐VEGF (1:1000, Santa Cruz), and anti‐BDNF (1:1000, Santa Cruz) overnight at 4°C, followed by treating with HRP‐conjugated secondary antibodies for 2 h. Blots were treated with ECL Detection Reagents (Millipore).

### 2.14 Statistical analysis

All data are presented as mean ± SEM, and all statistical analyses were two‐tailed with SPSS 22.0 Statistics. The Shapiro–Wilk test was used to analyze the normality of data. For normally distributed data, a one‐way analysis of variance was conducted, followed by the Bonferroni test if there were more than two groups. Nonparametric Kruskal–Wallis *H* test was performed to analyze the results from the mNSS and the corner test. Differences in survival rate between the groups were analyzed using the log‐rank (Mantel–Cox) test. *p* < 0.05 were considered statistically significant.

## 3 RESULTS

### 3.1 Identification of NSCs

**FIGURE 1:**
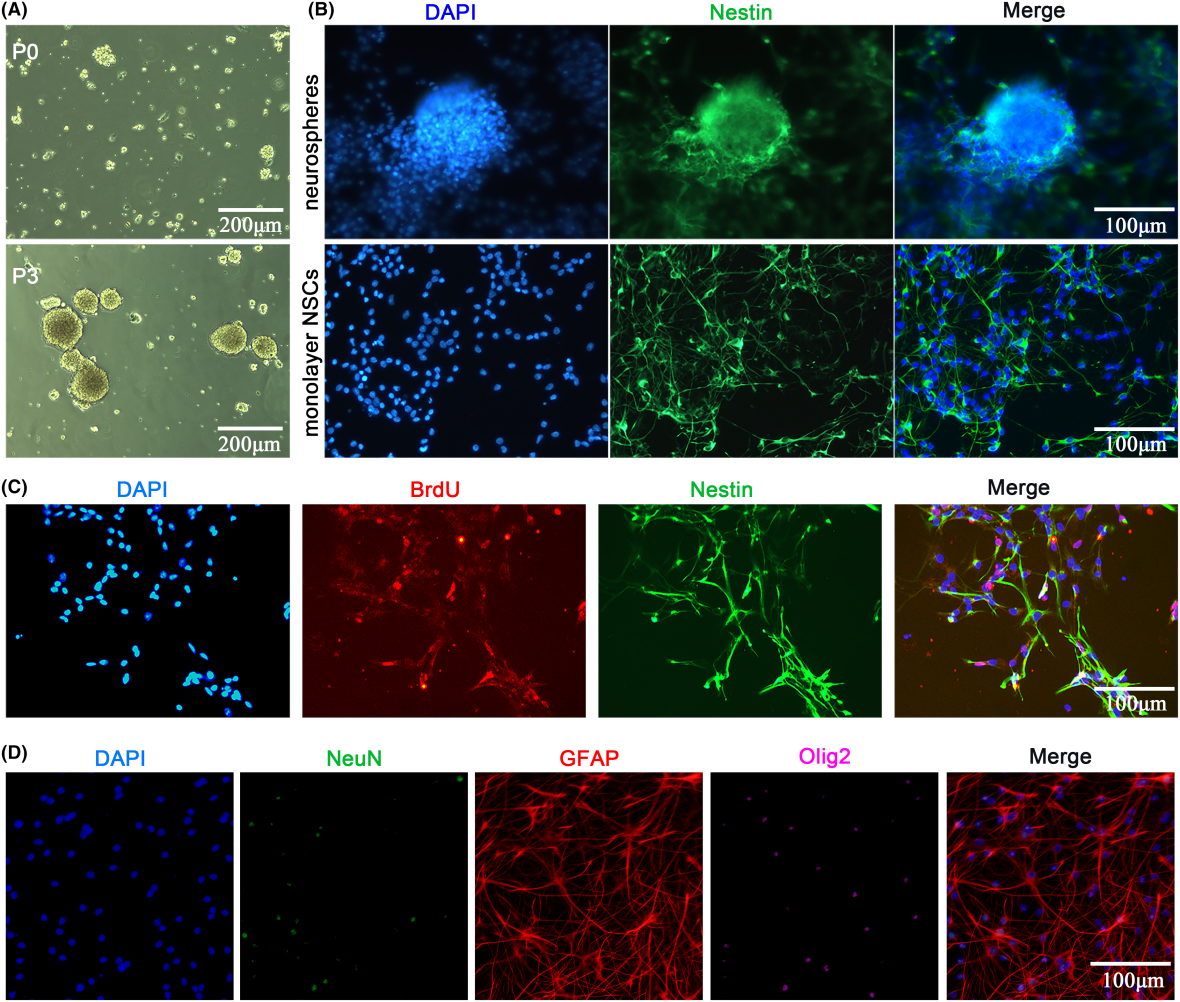
Identification of NSCs. (A) Morphology of primary cultured NSCs passage 0 (upper panel) and day 3 of passage 3 (lower panel). Scale bar = 200 μm. (B) Immunofluorescence staining of NSC marker. Cells were positive for Nestin in neurospheres and monolayer NSCs cultured in PDL‐coated well plates. Scale bar = 100 μm. (C) Representative immunofluorescence staining of BrdU (red) and Nestin (green) with nucleus (blue). Scale bar = 100 μm. (D) Representative immunofluorescence staining of NeuN (green), GFAP (red), and Olig2 (purple) to identify multipotency of the primary cultured NSCs. Scale bar = 100 μm.

The primary NSCs separated from the cerebral cortex of fetal rats rapidly proliferated and gave rise to free‐floating small clusters (neurospheres) after 48 h. The clusters continued to grow in size during expansion (Figure 1A). After 3 passages of expansion, the purity of NSCs was verified up to 99.12% by immunofluorescence staining of biomarker Nestin (a marker for NSCs) both in neurospheres and monolayer NSCs (Figure 1B). Meanwhile, the BrdU labeling was applied to examine the proliferation of NSCs. It was shown that approximately 20.83% of NSCs were dual‐positive staining of BrdU/Nestin (Figure 1C). After 7 days of differentiation, 19.05% of NSCs were differentiated into NeuN^+^ cells (a marker for neurons, Figure 1D), while 63.09% of NSCs into GFAP^+^ cells (a marker for astrocytes, Figure 1D), and 17.86% of NSCs into Olig2^+^ cells (a marker for oligodendrocytes, Figure 1D). These results demonstrated that the cultured NSCs were capable of further proliferation and differentiation.

### 3.2 miR‐199a‐5p promoted neuronal differentiation of NSCs

**FIGURE 2:**
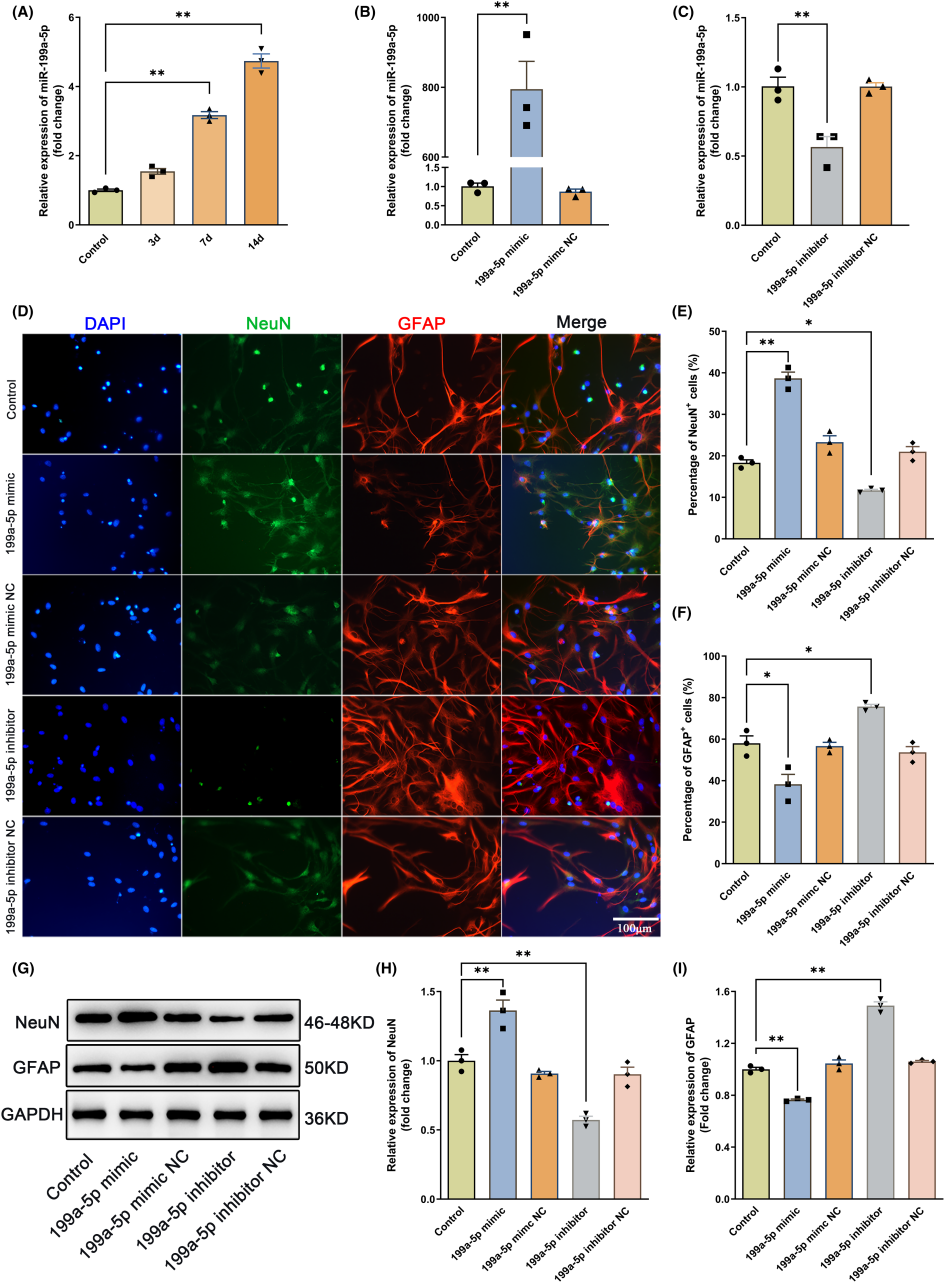
miR‐199a‐5p promoted neuronal differentiation of NSCs. (A) The expression of miR‐199a‐5p during the differentiation of primary cultured NSCs at 3 days, 7 days, and 14 days was evaluated by RT‐qPCR. *n* = 3. (B, C) The NSCs were transfected with miR‐199a‐5p mimic or inhibitor confirmed by RT‐qPCR. *n* = 3. (D) Representative images of double‐labeling immunostaining of NeuN^+^/GFAP^+^ in NSCs after transfected with miR‐199a‐5p mimic/NC or inhibitor/NC. Scale bar = 100 μm, *n* = 3. (E, F) Quantitative analysis of the percentage of NeuN^+^ and GFAP^+^ cells. (G–I) Western blots and semiquantitative analysis of NeuN and GFAP expression. *n* = 3. The data were presented as the mean ± SEM, *p < 0.05, and **p < 0.01.

To determine the function of miR‐199a‐5p on the differentiation of NSCs, we first measured the expression of miR‐199a‐5p by RT‐qPCR at the 3rd, 7th, and 14th d during NSCs differentiation. The involvement of miR‐199a‐5p in the differentiation of NSCs can be indicated by its gradually rising expression over time during NSC differentiation (Figure 2A). Both gain‐of‐function and loss‐of‐function approaches indicated a dramatically enhanced expression of miR‐199a‐5p in the miR‐199a‐5p mimic group, whereas decreased expression of miR‐199a‐5p in the miR‐199a‐5p inhibitor group (Figure 2B,C). Furthermore, the percentage of NeuN^+^ cells was significantly enhanced after the treatment of miR‐199a‐5p mimic, while those of GFAP‐positive cells decreased (Figure 2D–F). In contrast, repressing miR‐199a‐5p by transfection with its specific inhibitor exerted the opposite effects (Figure 2D–F). The Western blotting results were similar to those obtained from the immunofluorescence staining (Figure 2G–I). Collectively, the above observations demonstrated that miR‐199a‐5p promoted the differentiation of NSCs into neurons and inhibited the differentiation into astrocytes.

### 3.3 Cav‐1 is a direct target of miR‐199a‐5p

**FIGURE 3:**
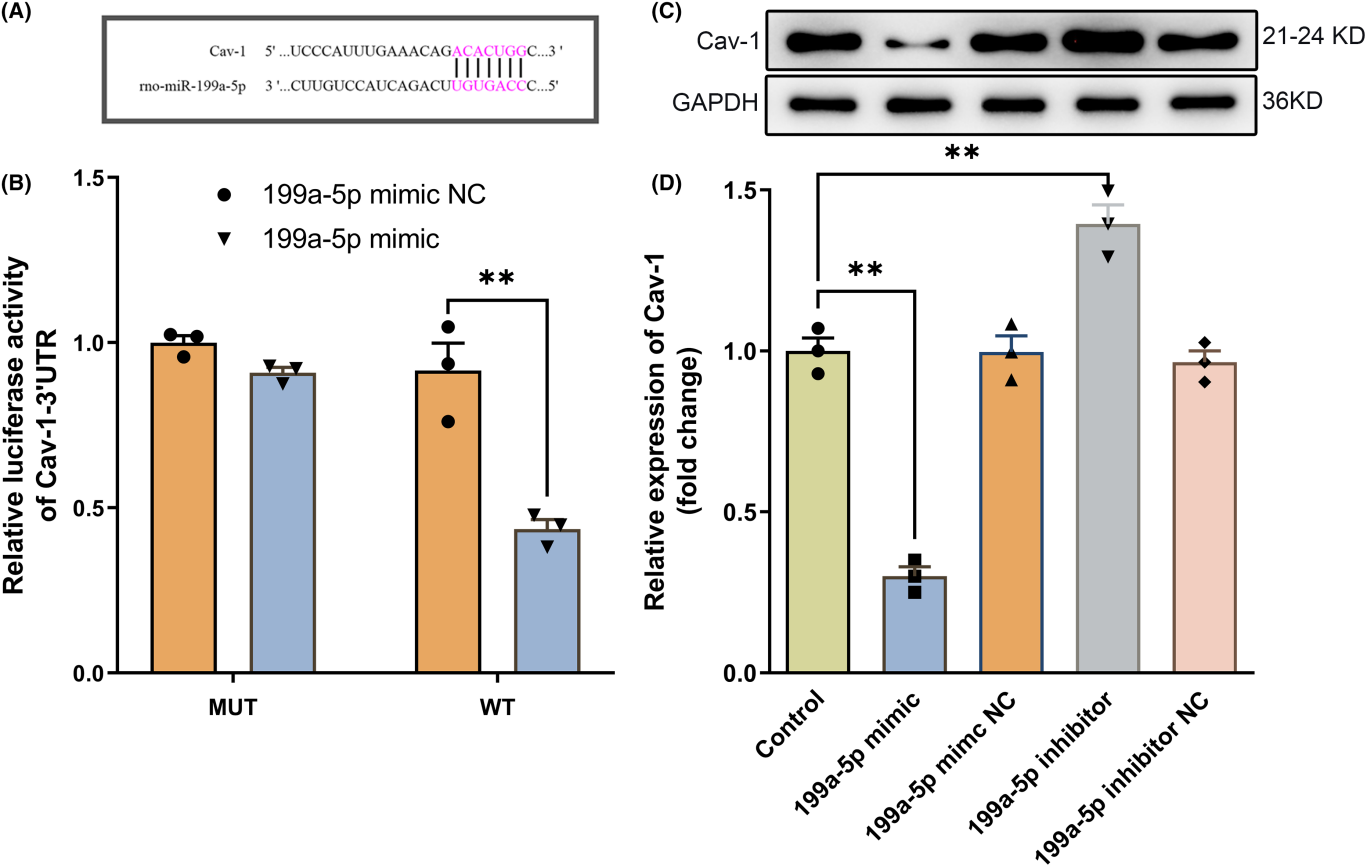
Cav‐1 is a direct target of miR‐199a‐5p. (A) Sequence alignment of Cav‐1 3′‐UTR with potential miR‐199a‐5p targeting sites identified via an online bioinformatic database. (B) Relative luciferase activity of the Cav‐1 ‐3'UTR WT or Cav‐1‐3'UTR MUT reporter co‐transfected with miR‐199a‐5p mimic or mimic NC. (C, D) Western blots and semiquantitative analysis of Cav‐1 expressions in NSCs transfected with miR‐199a‐5p mimic/NC or inhibitor/NC. *n* = 3. The data were presented as the mean ± SEM **p < 0.01.

To explore the mechanism by which miR‐199a‐5p modulated endogenous neurogenesis, we investigated the potential regulatory network of miR‐199a‐5p. The binding sites of miR‐199a‐5p and Cav‐1 were predicted by TargetScan (http://targetscan.org/), which found a binding site between miR‐199a‐5p and the 3′‐UTR of the Cav‐1 mRNA (Figure 3A). Furthermore, we performed the dual‐luciferase reporter assay to verify the predicted results. After the co‐transfection with the Cav‐1‐WT and miR‐199a‐5p mimic, luciferase activity remarkably decreased to 43.57% (Figure 3B) compared to the negative control. However, fluorescence intensity was not impacted by the co‐transfection with the Cav‐1‐MUT and miR‐199a‐5p mimic or the mimic NC (Figure 3B). Moreover, Western blotting revealed that the miR‐199a‐5p mimic significantly inhibited the expression of Cav‐1 (Figure 3C,D). However, the miR‐199a‐5p inhibitor increased the expression of Cav‐1 (Figure 3C,D).

### 3.4 Cav‐1 was involved in miR‐199a‐5p‐mediated NSCs differentiation in vitro

**FIGURE 4:**
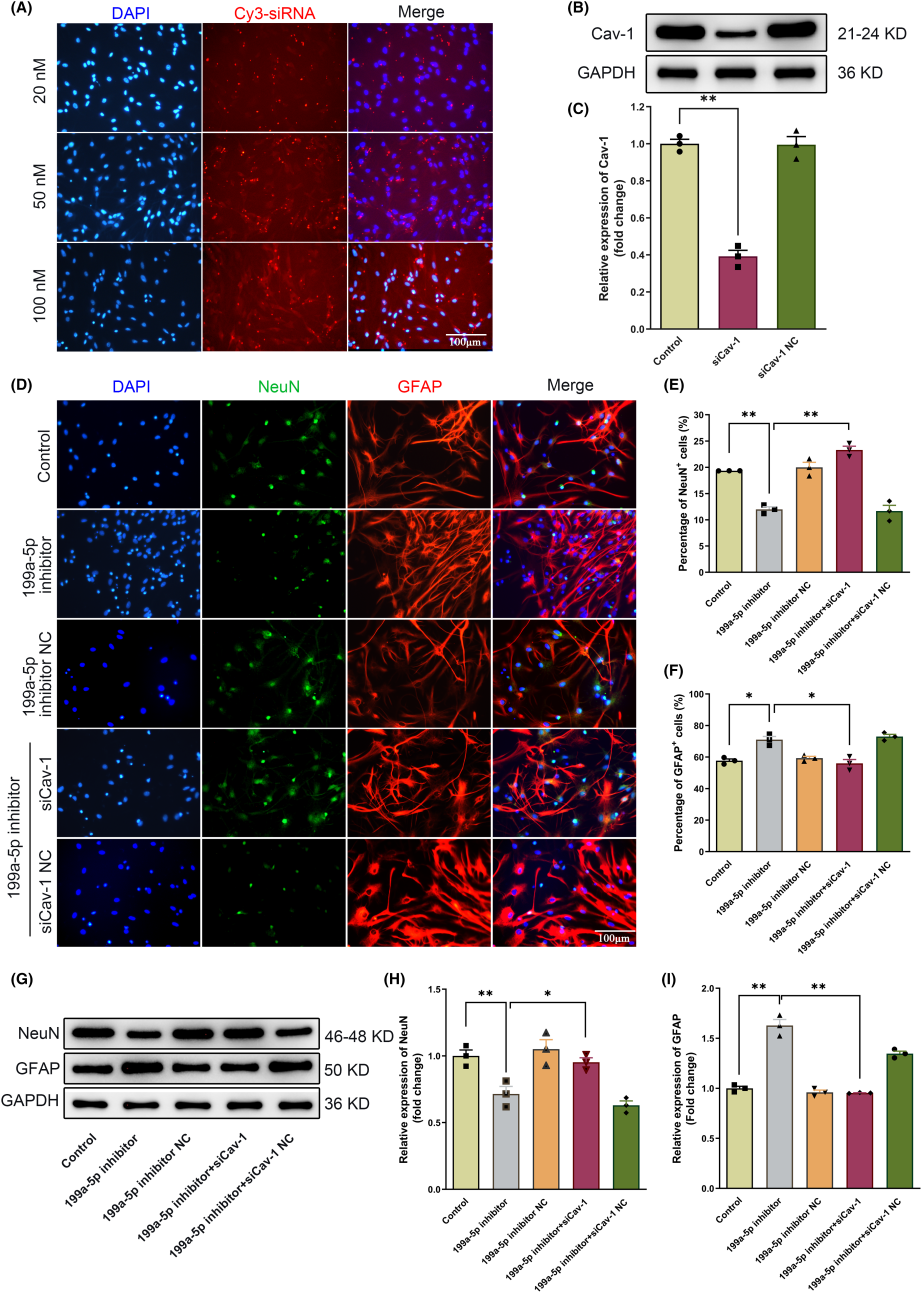
Cav‐1 was involved in miR‐199a‐5p‐mediated differentiation of NSCs in vitro. (A–C) NSCs were transfected with different concentrations (20 nM, 50 nM, and 100 nM) of Cav‐1 siRNA and verified by fluorescence and western blot. *n* = 3. (D–F) Representative images of double‐labeling immunostaining of NeuN and GFAP in NSCs after cotransfected with miR‐199a‐5p inhibitor/siCav‐1 or the corresponding negative control. Scale bar = 100 μm, *n* = 3. (G–I) Western blot and semiquantitative analysis of the NeuN and GFAP protein expression in NSCs transfected with miR‐199a‐5p inhibitor/siCav‐1 or the corresponding negative control. *n* = 3. The data were presented as the mean ± SEM, *p < 0.05, and **p < 0.01.

To find out the effects of Cav‐1 on NSCs differentiation, NSCs were transfected with different concentrations (20 nM, 50 nM, and 100 nM) of Cy3‐conjugated Cav‐1 siRNA. The results showed a highest transfection efficiency of 100 nM Cav‐1 siRNA (Figure 4A). Dramatical inhibition of Cav‐1 protein by Cav‐1 siRNA was also seen from the western blot results (100 nM, Figure 4B,C). Next, after cotransfection with miR‐199a‐5p inhibitor (100 nM) and Cav‐1 siRNA (100 nM), immunofluorescence staining demonstrated a reduction in the ratio of NeuN^+^ cells and enhancement in the ratio of GFAP^+^ cells by the treatment of miR‐199a‐5p inhibitor (Figure 4D–F). However, these effects were reversed as Cav‐1 was concurrently silenced (Figure 4D–F). In addition, the protein expression of NeuN and GFAP was further confirmed by Western blotting (Figure 4G–I). These results indicated that miR‐199a‐5p promoted neuronal differentiation of NSCs by downregulating Cav‐1.

### 3.5 miR‐199a‐5p improved neurological function recovery and reduced infarct volume after ischemic stroke

**FIGURE 5:**
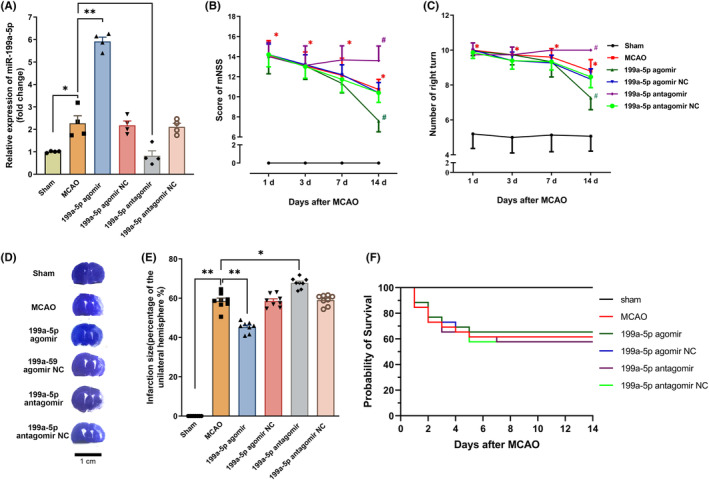
miR‐199a‐5p improved neurological function recovery and reduced infarct volume after ischemic stroke. (A) The expression of miR‐199a‐5p was determined by RT‐qPCR. *n* = 4, *p < 0.05, **p < 0.01. (B, C) mNSS and the corner test. *n* = 15, *p < 0.05 vs sham group, ^#^ *p* < 0.05 versus MCAO group. (D) Representative images of brain sections by toluidine blue staining. (E) Quantification of infarct volume at 14th d after MCAO. *n* = 8. *p < 0.05, and **p < 0.01. The data were presented as the mean ± SEM. (F) Fourteen‐day survival rates of each group after cerebral ischemia. *n* = 15–17.

To determine the protection of miR‐199a‐5p on neurological function recovery, miR‐199a‐5p agomir/NC and miR‐199a‐5p antagomir/NC were delivered via intracerebroventricular injection. RT‐qPCR results showed that the miR‐199a‐5p expression was significantly increased at 14th d after ischemia compared with the sham group (Figure 5A). In addition, miR‐199a‐5p expression was strikingly augmented after the treatment of miR‐199a‐5p agomir, which was slightly suppressed by antagomir administration (Figure 5A). The mNSS and corner test were performed on days 1, 3, 7, and 14 after ischemia. Administration of the miR‐199a‐5p agomir could improve the neurological deficits at 14th d on the mNSS scores and corner test (Figure 5B,C), whereas miR‐199a‐5p antagomir hampered the recovery of neurological function (Figure 5B,C). Moreover, the administration of miR‐199a‐5p agomir significantly reduced infarct volume at the 14th d after ischemia compared with the MCAO group (Figure 5D,E). Conversely, miR‐199a‐5p antagomir increased infarct volume (Figure 5D,E). Additionally, there was no significant difference in the survival rate at the 14th day between the MCAO group (61.5%, 16/26) and miR‐199a‐5p agomir group (65.4%, 17/26), miR‐199a‐5p agomir NC group (65.4%, 17/26), miR‐199a‐5p antagomir group (57.7%, 15/26) or miR‐199a‐5p antagomir NC group (57.7%, 15/26, Figure 5F).

### 3.6 miR‐199a‐5p promoted endogenous neurogenesis in the peri‐infarct regions after ischemia in rats

**FIGURE 6:**
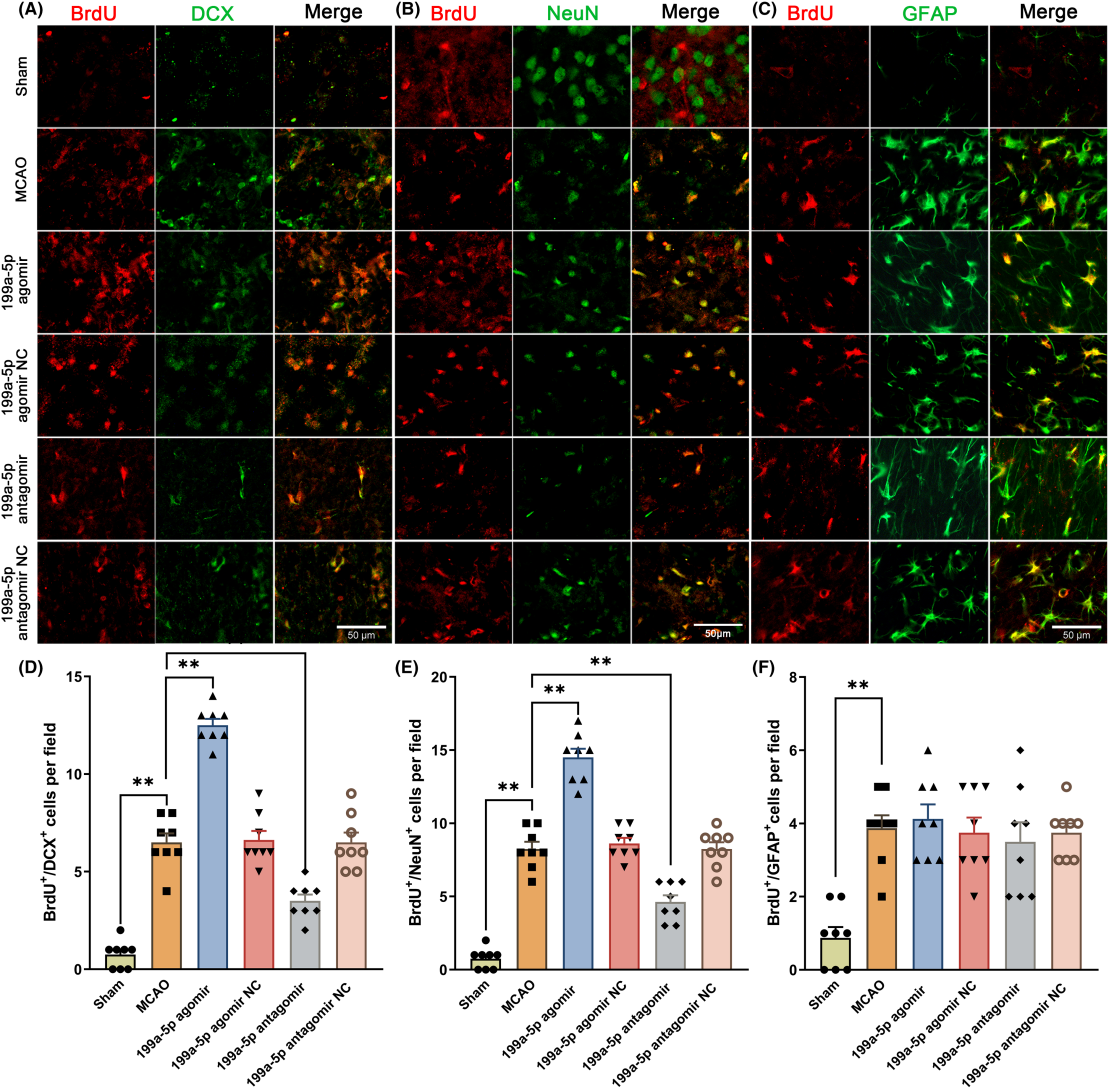
miR‐199a‐5p promoted endogenous neurogenesis in the peri‐infarct regions after ischemia in rats. (A–C) Representative images of BrdU/DCX, BrdU/NeuN, and BrdU/GFAP double immunofluorescence staining in the peri‐infarct regions after ischemic stroke in rats. (D–F) Quantification of the number of BrdU^+^/DCX^+^, BrdU^+^/NeuN^+^ and BrdU^+^/GFAP^+^ cells in the peri‐infarct regions. Scale bar = 50 μm, the data were presented as the mean ± SEM, *n* = 8, and **p < 0.01.

We further explored the function of miR‐199a‐5p on the post‐stroke NSCs differentiation at 14th d. BrdU^+^/DCX^+^ cells in the MCAO group were higher than their counterparts in the sham group (Figure 6A,D). After the treatment of miR‐199a‐5p agomir, the number of BrdU^+^/DCX^+^ cells further elevated compared with that of the MCAO group (Figure 6A,D). Moreover, BrdU^+^/NeuN^+^ cells greatly raised in the miR‐199a‐5p agomir group and reduced in the miR‐199a‐5p antagomir group in comparison with the MCAO group (Figure 6B,E). Neither the treatment of the miR‐199a‐5p agomir nor miR‐199a‐5p antagomir produced any significant effect on BrdU^+^/GFAP^+^ cells (Figure 6C,F). These results demonstrated that miR‐199a‐5p promoted endogenous neurogenesis in rats after cerebral ischemia.

### 3.7 miR‐199a‐5p promoted endogenous neurogenesis through inhibiting Cav‐1

**FIGURE 7:**
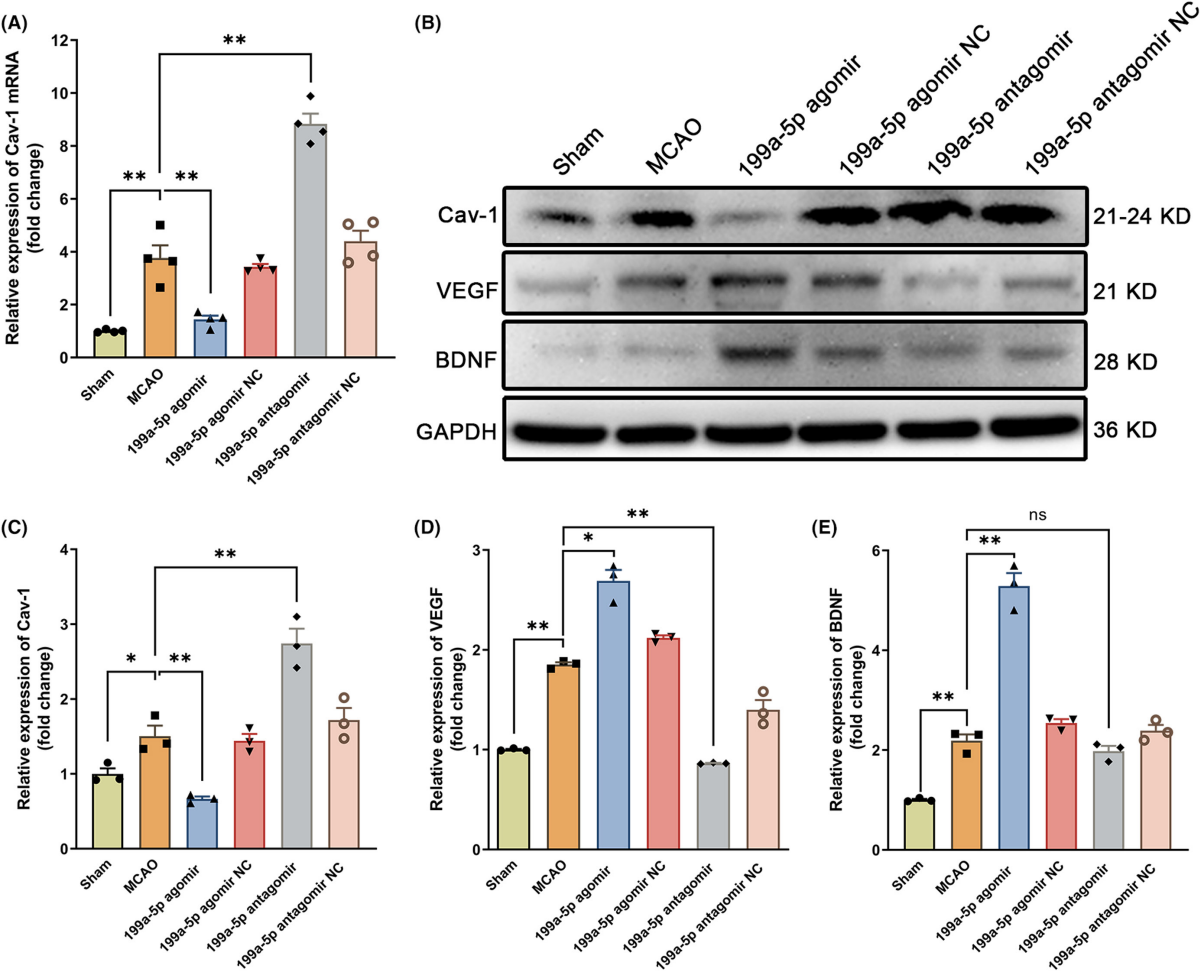
miR‐199a‐5p promoted endogenous neurogenesis through inhibiting Cav‐1 and increasing VEGF and BDNF expression. (A) The mRNA expression levels of Cav‐1 were determined by RT‐qPCR at 14th d after ischemia. *n* = 4. (B) Representative bands of Cav‐1, VEGF, and BDNF expressions by western blot. (C–E) Semiquantitative analysis of the Cav‐1, VEGF, and BDNF proteins. The data were presented as the mean ± SEM, *n* = 3. *p < 0.05, and **p < 0.01.

Both RT‐qPCR and western blot results showed that the forced expression of miR‐199a‐5p by miR‐199a‐5p agomir contributed to the decreased expression of Cav‐1 (Figure 7A–C). Administration of miR‐199a‐5p antagomir facilitated the expression of Cav‐1 at 14th d after ischemia (Figure 7A–C). The above findings suggested that the miR‐199a‐5p boosted endogenous neurogenesis by negatively modulating the Cav‐1 target gene.

Subsequently, the expression of vascular endothelial growth factor (VEGF) and brain‐derived neurotrophic factor (BDNF) were measured, which are key neurotrophic factors for neurogenesis after ischemic stroke. MiR‐199a‐5p agomir notably elevated the expression levels of VEGF and BDNF (Figure 7B,D,E). In contrast, the administration of miR‐199a‐5p antagomir decreased their expressions compared with the MCAO group (Figure 7B,D,E).

## 4 DISCUSSION

That ischemic stroke often leads to long‐term neurological deficits, with no specific therapeutic options available for late recovery currently has become a major concern.^30^ We demonstrate in the current study that miR‐199a‐5p by downregulating Cav‐1 in vitro motivated neuronal differentiation and suppressed glial differentiation of NSCs. Moreover, the in vivo study of miR‐199a‐5p showed in rats its enhancing endogenous neurogenesis and improving functional outcomes after focal cerebral ischemia. These data suggested that miR‐199a‐5p improve the recovery of neural function by promoting neuronal differentiation of NSCs after ischemic stroke.

miRNAs participation in the occurrence and progression of ischemic stroke in animals and patients have been confirmed by either miRNA expression profile or sequencing from multiple research.^31^, ^32^ In particular, the effect of miR‐199a‐5p on ischemia‐induced neural plasticity has been widely studied.^33^, ^34^ Expression of miR‐199a‐5p was explicitly found in nerve tissue, with its biofunctions involving the remodeling of the neurovascular unit and synaptic plasticity.^33^, ^34^ miR‐199a‐5p also preserves against hypoxia‐induced cerebral injury, spinal cord injury, and myocardial injury.^16^, ^35^, ^36^ Nevertheless, the role of miR‐199a‐5p during the post‐ischemic neurogenesis had not been determined.

NSCs as pluripotent stem cells with self‐renewal ability can differentiate into neurons, astrocytes, and oligodendrocytes.^9^ Focal cerebral ischemia can motivate endogenous neurogenesis to improve functional recovery by promoting NSC proliferation, and differentiation into new neurons, as well as their migration to damaged brain areas.^11^ In our study, we verified the vital function of miR‐199a‐5p in regulating endogenous neurogenesis. DCX^+^ neuroblasts are usually identified as migrating neuroblasts that already possess neuronal commitment.^9^ An increase of BrdU^+^/DCX^+^ cells was observed following the administration of miR‐199a‐5p agomir, whereas the number of BrdU^+^/DCX^+^ cells significantly decreased with the treatment of miR‐199a‐5p antagomir. Thus, these findings indicate that miR‐199a‐5p after stroke promotes the proliferation and recruitment of SVZ NSCs in rats.

Under pathophysiological conditions, most NSCs eventually differentiate into astrocytes which in the injured brain can be transformed into reactive astrocytes and promote the formation of glial scars, thereby inhibiting axon regeneration and hindering neurogenesis.^13^, ^37^ In this study, we found that the expression of miR‐199a‐5p elevated at 14th d after cerebral ischemia. After transfected with miR‐199a‐5p mimic, NSCs enhanced neuronal differentiation and weakened astrocyte differentiation in vitro. However, this effect was reversed by the miR‐199a‐5p inhibitor. More importantly, miR‐199a‐5p promoted neuronal differentiation of NSCs in the peri‐infarct regions in the rat model of ischemic stroke, as confirmed by double labeling with BrdU and NeuN. Notably, the miR‐199a‐5p agomir did not affect the number of BrdU^+^/GFAP^+^ cells in vivo. Collectively, miR‐199a‐5p did change the differentiation fate of NSCs, leading to increased neurogenesis and decreased gliogenesis.

The predicted target genes of miR‐199a‐5p showed a corresponding binding site with Cav‐1 at the 3′UTR. A dual‐luciferase reporter assay apart from bioinformatic analysis was performed and further confirmed the relationship between Cav‐1 and miR‐199a‐5p, which was consistent with the results of previous studies.^18^, ^38^ The inhibited Cav‐1 protein upon overexpression of miR‐199a‐5p and increased Cav‐1 induced by miR‐199a‐5p repression suggested that Cav‐1 in NSCs be a target gene of miR‐199a‐5p. Cav‐1 refers to a small spherical invaginated plasma membrane of 50–100 nm. Li et al. have reported that Cav‐1 inhibited neuronal differentiation and promoted astroglial differentiation of neural stem/progenitor cells and may also be a key participant in regulating neurogenesis.^19^, ^20^, ^21^ Studies have shown that Cav‐1 inhibited the differentiation of NSCs into neurons by regulating VEGF and BDNF,^19^, ^20^, ^21^ both of which were considered the most potent and specific angiogenesis and neurogenesis factors.^39^, ^40^ In the present study, we found that the down‐regulation of Cav‐1 following the treatment of miR‐199a‐5p agomir was accompanied by the increased expression of VEGF and BDNF after ischemic stroke in rats. In contrast, the elevation of Cav‐1 following the treatment of miR‐199a‐5p antagomir was simultaneously along with lower expression of VEGF and BDNF. These results suggest that the low expression of Cav‐1 induced by miR‐199a‐5p contributes to the increased expression of BDNF and VEGF, eventually altering the differentiation fate of NSCs and promoting neurogenesis.

Although this study presented evidence that miR‐199a‐5p promoted neuronal differentiation of neural stem cells and endogenous neurogenesis after ischemic stroke, there were certain limitations. First, previous reports showed the involvement of a large number of miRNAs in the pathophysiological processes of stroke, in the present study however, due to the major focus on exploring the role of miR‐199a‐5p, the remaining miRNAs that might also contribute to long‐term outcomes of ischemic nerve repair were not included, but will further be taken into consideration in the following research. Second, it is unsurprising that a single miRNA may modulate a series of transcripts, and various miRNAs can modulate the same mRNA as well. Multiple target genes of miR‐199a‐5p have been identified, such as CDKN1B and Sirtuin‐1.^41^, ^42^ Thus, we cannot rule out the possibility of the participation of potential targets other than Cav‐1 in miR‐199a‐5p‐mediated endogenous neurogenesis.

## 5 CONCLUSIONS

Results of the present study supported a critical role of miR‐199a‐5p by inhibiting Cav‐1 expression to promote the neuronal differentiation of NSCs and endogenous neurogenesis in rats after cerebral ischemia. These findings further suggest that miR‐199a‐5p/Cav‐1 pathway be a novel therapeutic approach for the postischemic stroke functional recovery.

## AUTHOR CONTRIBUTIONS

Hua‐qian Jin, Wei‐feng Jiang, Xin‐tian Zheng, and Lin Li conceived and designed the study, performed the in‐vivo experiments, analyzed the data and wrote the manuscript. Xin‐tian Zheng, Yan Fang, Yan Yang, and Xiao‐wei Hu performed in‐vitro experiments and analyzed the data. Li‐sheng Chu contributed to the study design, data analysis and interpretation, and manuscript writing and revision. All authors read and approved the manuscript.

## CONFLICT OF INTEREST STATEMENT

The authors declare that they have no competing interests.

## Data Availability

The data that support the findings of this study are available from the corresponding author upon reasonable request.
